# Ceftriaxone-Induced Immune Hemolytic Anemia

**DOI:** 10.7759/cureus.8660

**Published:** 2020-06-17

**Authors:** Anil Singh, Namrata Singhania, Amit Sharma, Namita Sharma, Subhankar Samal

**Affiliations:** 1 Hospital Medicine, Geisinger Community Medical Center, Scranton, USA; 2 Hospital Medicine, Mount Carmel Hospital, Columbus, USA; 3 Infectious Disease, Geisinger Community Medical Center, Scranton, USA; 4 Hematology, Geisinger Community Medical Center, Scranton, USA; 5 Internal Medicine, Ascension Health, Milwaukee, USA

**Keywords:** ceftriaxone, immune hemolytic anemia

## Abstract

Ceftriaxone is a commonly used antibiotic in hospitals for the treatment of pneumonia, urinary tract infection, bacteremia, meningitis, skin, and soft tissue infection. It can be associated with common allergic reactions like skin rash, itching, and, rarely, angioedema. Ceftriaxone-induced immune hemolytic anemia (IHA) is a rare and potentially fatal complication if not identified and managed in time. We report a case of ceftriaxone-induced IHA in a young woman.

## Introduction

Drug-induced immune hemolytic anemia (IHA) is an uncommon cause of hemolytic anemia [1]. Several drugs can cause IHA, including antibiotics (penicillin or cephalosporins) and anticancer medications [2-3]. The reaction is mainly immune complex-mediated and can sometimes be severe and life-threatening [4-5]. Herein, we report a case of severe ceftriaxone-induced IHA requiring drug discontinuation, blood transfusion, and supportive care.

## Case presentation

A 35-year-old woman with a past medical history of diabetes mellitus and hypertension was initially admitted to the hospital with a nonhealing ulcer on the left foot and was diagnosed with osteomyelitis of the left foot. She was discharged to home on IV ceftriaxone for a total of six weeks. After discharge, she experienced low back pain with IV doses of ceftriaxone. An infectious disease specialist advised her to slow the rate of administration. Still, her symptoms did not resolve and eventually progressed to band-like chest pains for one hour during and after IV ceftriaxone administration. She also had associated nausea, diaphoresis, and dizziness. She took three doses of IV ceftriaxone at home before she came back to the hospital and was admitted for the above-mentioned symptoms.

Her vitals were stable except for tachycardia (heart rate: 105 beats per minute). Her physical examination findings were unremarkable. Laboratory indices showed her hemoglobin (Hb) was 6.1 g/dL (baseline: 9.5 g/dL four days prior), her white blood cell (WBC) count was 42,430/mm3, and her platelet count was 595,000/mm3. Her blood urea nitrogen (BUN) was 20 mg/dL, creatinine was 1.5 mg/dL, sodium was 137 mmol/L, potassium was 4.8 mmol/L, total bilirubin was 2.0 mg/dL (baseline: normal), direct bilirubin was 0.5 mg/dL, lactate dehydrogenase (LDH) was 1075 U/L, haptoglobin was < 10 mg/dL, lactic acid was 3.0 mmol/L, and reticulocytes were 3.30%. The summary of relevant laboratory indices is shown in Table 1.

**Table 1:** Summary of relevant laboratory indices at admission. L, low; H, high; HH, very high; LL, very low BUN, blood urea nitrogen; eGFR, estimated glomerular filtration rate; WBC, white blood cell count; Hb, hemoglobin; Hct, hematocrit; MCV, mean corpuscular volume; MCH, mean corpuscular hemoglobin; MCHC, mean corpuscular hemoglobin concentration; AST, aspartate aminotransferase; ALT, alanine aminotransferase; LDH, lactate dehydrogenase

Analyte	Reference range	Laboratory value
BUN	6-20 mg/dL	20
Creatinine	0.5-1.0 mg/dL	1.5 (H)
Sodium	135-146 mmol/L	137
Potassium	3.5-5.1 mmol/L	4.8
Bicarbonate	22-32 mmol/L	25
Anion gap	7-15 mmol/L	11
Glucose	70-120 mg/dL	295 (H)
Calcium	8.4-10.2 mg/dL	8.5
eGFR	> 60	44.9 (L)
WBC	4.00-10.80 K/uL	42.43 (HH)
Hb	12.0-15.3 g/dL	6.1 (LL)
Hct	36.0%-45.2%	17.1 (L)
MCV	81.5-97.5 fL	92.9
MCH	27.0-34.0 pg	33.2
MCHC	32.0-36.0 g/dL	35.7
Platelet count	140-400 K/uL	595 (H)
Neutrophils	40%-75%	72.5
Lymphocytes	18%-42%	12.8 (L)
Troponin I	< 0.04 ng/mL	< 0.030
Total bilirubin	0-1.2 mg/dL	2.0
Direct bilirubin	0-0.3 mg/dL	0.5
AST	10-35 U/L	217
ALT	10-35 U/L	67
Alkaline phosphatase	0-153 U/L	72
Albumin	3.8-5.0 g/dL	2.9
Total protein	6.0-8.3 g/dL	7.3
Lactic acid	0.4-2.5 mmol/L	3.0 (H)
LDH	90-250 U/L	1,075 (H)
Reticulocyte%	0.80%-1.9%	3.30 (H)
Haptoglobin	30-200 mg/dL	< 10 (L)

Peripheral blood smear showed with occasional spherocytes. Based on the above laboratory findings, drug-induced hemolytic anemia secondary to ceftriaxone was suspected. Direct Coombs (direct antiglobulin test; DAT) was positive for immunoglobin (Ig) G and complement C3. A chest radiograph was unremarkable. An electrocardiogram showed sinus tachycardia with a heart rate of 105 beats per minute and nonspecific ST-T changes. A CT scan of the abdomen and pelvis showed moderate splenomegaly.

Ceftriaxone was discontinued, and she was started on vancomycin and piperacillin/tazobactam. She was also given a blood transfusion. Her symptoms and laboratory indices, including hemoglobin and WBC count, began to improve soon after hospitalization. Blood cultures and urine cultures that were taken on the day of admission came back negative, and antibiotics were de-escalated. At discharge, she was switched to IV ampicillin/sulbactam to complete a total of six weeks’ course of antibiotics. After that, she remained asymptomatic throughout her antibiotic course.

## Discussion

Ceftriaxone is a third-generation cephalosporin commonly used in an inpatient setting for the treatment of multiple infections like urinary tract infection and community-acquired pneumonia. Drug-induced hemolytic anemia is not a common cause of hemolytic anemia. Various antibiotics, such as ceftriaxone, can cause it. A study found that of 73 patients with drug-induced IHA, 16% were due to ceftriaxone [6]. Another report found cephalosporins to be a cause of IHA in approximately 50% of patients [2].

The primary mechanism of drug-induced IHA is due to immune destruction of red blood cells (RBCs). This is antibody-mediated, where antibody-bound RBCs are phagocytized by macrophages in the spleen, leading to hemolysis [4]. It can be classified into two different pathways: drug-dependent (more common) and drug independent. The drug-dependent or hapten pathway of reactions can be further classified into two different types: penicillin or nonpenicillin reaction. In the penicillin reaction, the drug binds to the RBC surface required for antibody binding. In the nonpenicillin response, the presence of the drug on the RBC surface is not required for antibody binding. In the drug-independent pathway, a change in the normal membrane component of RBCs can occur, which attracts antibodies and causes immune complex formation.

Drug-induced IHA is usually seen within days to weeks of exposure to new drugs. The first step in detecting this adverse reaction is to identify symptoms. Common symptoms are fatigue, jaundice, dyspnea, abdominal pain, or low back pain. Most of the symptoms are related to hemolysis. The next step is to order appropriate laboratory tests, as mentioned above, along with a peripheral blood smear. Common abnormalities associated with hemolysis include low hemoglobin, high levels of LDH, low haptoglobin, and high indirect bilirubin. A peripheral blood smear can show bite cells, spherocytes, or schistocytes (as in thrombotic microangiopathy). After this, ordering DAT is useful. Reports have demonstrated that DAT in cephalosporin-induced IHA is positive for anti-C3 antibodies in 100% of patients and anti-immunoglobulin G (IgG) in 47% of patients [5]. These antibodies are primarily immunoglobulin M (IgM). DAT in penicillin-induced IHA can be negative for anti-C3.

The management of drug-induced IHA is based on the severity of symptoms and hemolysis. Reactions in children are usually severe. In one report, 36% of patients had fatal drug-induced IHA [5]. Hence, early identification and treatment are vital. Discontinuation of the presumed offending drug is the first line of treatment. Most mild to moderate cases significantly improve with discontinuation. Patients with severe, symptomatic anemia (hemoglobin levels < 7 g/dL) require blood transfusion. Rarely, the patient requires an exchange transfusion in cases of shock. Transfusion should not be withheld in patients with severe anemia, although it may be difficult to locate crossmatch-compatible blood in patients with a positive DAT. Patients can also develop severe complications from intravascular hemolysis, including acute kidney injury, disseminated intravascular coagulation, or thrombotic microangiopathy. Autoantibodies due to ceftriaxone are drug-dependent, and hemolysis usually resolves once the drug is stopped. The antibodies can still be detected for the next one to two weeks, but they usually do not cause hemolysis unless the patient is exposed to the drug again.

Our patient had ceftriaxone-induced IHA. Her symptoms and laboratory indices significantly improved after discontinuation of ceftriaxone. She required packed RBCs transfusion for the first three days, but hemoglobin remained stable afterward. Fortunately, she did not develop any hemolytic reactions to penicillin and tolerated piperacillin/tazobactam initially and ampicillin/sulbactam later.

## Conclusions

This case report highlights a rare but potentially fatal complication of cephalosporin-induced IHA. Early diagnosis and prompt treatment with discontinuation of the offending agent, blood transfusion, and supportive care was essential. We also recommend re-evaluating the status of autoantibodies if future transfusions are required.
